# A Mobile Health App (WYZ) for Engagement in Care and Antiretroviral Therapy Adherence Among Youth and Young Adults Living With HIV: Single-Arm Pilot Intervention Study

**DOI:** 10.2196/26861

**Published:** 2021-08-31

**Authors:** Parya Saberi, Nadra E Lisha, Xavier A Erguera, Estie Sid Hudes, Mallory O Johnson, Theodore Ruel, Torsten B Neilands

**Affiliations:** 1 Department of Medicine University of California, San Francisco San Francisco, CA United States; 2 Center for Tobacco Control Research and Education University of California, San Francisco San Francisco, CA United States; 3 Department of Pediatrics University of California, San Francisco San Francisco, CA United States

**Keywords:** youth living with HIV, mobile health, mobile app, engagement in care, antiretroviral therapy adherence, pilot

## Abstract

**Background:**

Youth are globally recognized as being vulnerable to HIV. Younger age has been correlated with worse health outcomes. Mobile health (mHealth) interventions have the potential to interact with youth where they are, using a device they already access.

**Objective:**

Using predefined benchmarks, we sought to evaluate the feasibility and acceptability of WYZ, an mHealth app, for improved engagement in care and antiretroviral therapy (ART) adherence among youth and young adults living with HIV. WYZ was designed and developed with input from youth and young adults living with HIV using a human-centered design approach and was based on the information, motivation, and behavioral skills framework to address common barriers to care and ART adherence among youth and young adults living with HIV.

**Methods:**

We recruited youth and young adults living with HIV (18-29 years old) from the San Francisco Bay Area to take part in a 6-month pilot trial. Their participation included completing baseline and exit surveys, and participating in seven phone check-ins about their use of WYZ.

**Results:**

Youth and young adults living with HIV (N=79) reported high levels of feasibility and acceptability with WYZ use. We met predefined benchmarks for recruitment (79/84, 94%), mean logins per week (5.3), tracking ART adherence (5442/9393, 57.9%), posting chat topics per week (4.8), and app crashes reported per week (0.24). The ease of app download, install, and setup, and comfort with security, privacy, and anonymity were highly rated (all over 91%). Additionally, participants reported high satisfaction for a research project that was remotely conducted. Participants used the app for shorter timeframes compared to the predefined benchmark.

**Conclusions:**

We noted high feasibility and acceptability with WYZ. Further research to examine the efficacy of WYZ will enable youth and young adults living with HIV and their providers to make informed decisions when using, recommending, and prescribing the app for improved engagement in HIV care and ART adherence.

**Trial Registration:**

ClinicalTrials.gov NCT03587857; https://clinicaltrials.gov/ct2/show/NCT03587857

## Introduction

In the United States, youth and young adults carry a significant burden of HIV. Youth and young adults living with HIV experience disparities at all steps of the HIV care continuum, including higher HIV incidence, lower linkage and retention in care, suboptimal antiretroviral therapy (ART) adherence, and lower virologic suppression [1-6]. The consequences of continued disparities include poor health outcomes, development and transmission of drug-resistant viruses, a future generation of adults who are more susceptible to developing AIDS, and further widening of these health disparities. Youth and young adults living with HIV experience many individual, structural (eg, transition to adult health care, inexperience with medical systems, and lack of insurance), social (eg, poverty, unstable housing, food insecurity, social isolation, and stigma), and biological (cognitive developmental stages) challenges that impact their abilities to access and adhere to oral ART [2,3,7-9]. However, there are few effective and tailored interventions that address ART adherence and engagement in HIV care for youth and young adults living with HIV.

In the United States, over 96% of youth and young adults living with HIV own smartphones [10], over two-thirds have downloaded mobile health (mHealth) apps [11], and over 90% are social media users [12]. The nearly ubiquitous access to and use of smartphones represents a powerful platform for the delivery of mHealth interventions to this population. Additionally, given the reduction in transportation costs, time constraints, potential stigma associated with participation in in-person HIV research [13], and missing data, mHealth technology can surmount common barriers, increasing the reach and generalizability of findings. Several mHealth apps are in various stages of development for people living with HIV [14-17], as we have previously summarized [18]. However, despite technology-based behavioral interventions showing promise in older adults living with HIV [19], few interventions have shown efficacy in addressing the unique aspects of youth developmental phases, youth culture, and gravitation of youth toward the use of technology [20]. In this study, we pilot tested an mHealth app to address barriers to engagement in care among youth and young adults living with HIV.

## Methods

### Study Design and Sample

From July 2019 to May 2020, we conducted a 6-month single-arm pilot study to evaluate the feasibility and acceptability of an mHealth app, named WYZ (pronounced “wise”), to address barriers to engagement in HIV care among individuals aged 18 to 29 years living with HIV in the San Francisco Bay Area [18]. WYZ was designed and developed using a human-centered design (HCD) approach [21-24]; formative research with youth and young adults living with HIV [18,20,25-27]; the information, motivation, and behavioral skills (IMB) [28-30] framework; and mHealth designers and developers from the University of California, San Francisco (UCSF) School of Medicine Technology team (SOM Tech). HCD focuses on creating approaches and delivering solutions to problems based on efforts to understand the specific needs and perspectives of the users. Therefore, HCD seeks to gain insights into the needs of the beneficiaries of an innovation, and creates approaches and delivers solutions to meet their needs.

Details of WYZ design and development, as well as the pilot study protocol, have previously been published [18]. In short, WYZ contains three main features, My Health, My Team, and My Community. My Health allows users to keep track of their ART medication information, visualize their adherence and laboratory data, and understand their health; My Team provides community resources and facilitates communication with health care team members; and My Community allows for social support from peers through anonymous and moderated discussion forums and allows users to stay up-to-date on health-related news. These features were developed with guidance from youth and young adults living with HIV and further refined through focus groups with youth and young adults living with HIV and iterative field testing with our Youth Advisory Panel (YAP), and were chosen to address specific barriers to ART adherence and engagement in HIV care (eg, social isolation and lack of community support).

WYZ design, development, and technological support were provided by UCSF’s SOM Tech. To ensure Health Insurance Portability and Accountability Act (HIPAA) compliance, we used Salesforce as the backend service and for storing sensitive data in a secure cloud-based database. Data about app usage were collected using Flurry (a mobile analytics tool) and Salesforce analytics. To enhance the security and privacy of WYZ, we used a two-step authentication process for downloading, password protection (with each log in), aliases, deletion of all communications over 30 days old, and remote revocation of app access in case of theft, loss, or misuse.

Participants were recruited using various strategies, including flyers at clinics and community-based organizations, emails to clinicians at clinics serving youth and young adults living with HIV, peer referral, and contacting prior study participants who had consented to being notified of future research. Information about the study was also disseminated through the YAP.

Individuals aged 18 to 29 years living with HIV, who lived or received medical care in the San Francisco Bay Area, spoke English, and had access to an Android or iOS smartphone, were included. Those with any evidence of severe cognitive impairment or active psychosis that impeded their ability to provide informed consent were excluded. To confirm an individual’s age and HIV serostatus, the potential participant text messaged a photo identification showing their date of birth and either a clinician’s letter of HIV diagnosis, a copy of laboratory test results (for HIV antibody or HIV viral load), or their ART medication vial. These photos were sent via text message to an encrypted and secure study phone for verification by study staff.

All study activities, including recruitment, screening, enrollment, study assessments, provision of incentives, and exit interviews, were conducted remotely using text message, telephone, email, and videoconference. Participants received a check-in at weeks 1, 2, and 4, followed by monthly check-ins, and up to US $215 for completion of all study activities. All procedures were reviewed and approved by the UCSF Institutional Review Board with a requirement for electronic consent. At baseline and 6 months, participants completed study assessments using a Qualtrics survey.

### Measures

#### Demographics

Demographic data, including date of birth, sex at birth, sexual identity, race/ethnicity, perceived financial security, and work status (full time, part time, or not working), were collected.

#### Feasibility Metrics

Feasibility metrics were collected using Flurry and Salesforce analytics. Metrics were based on predefined thresholds [18], including how many people were recruited for the study, mean logins to the app, mean minutes in the app, and use of specific features in the app.

#### Acceptability Metrics

Acceptability metrics were collected using a Qualtrics survey administered during the last study visit at 6 months. The survey included questions related to satisfaction with WYZ, ease of WYZ use, and satisfaction with the study. Additionally, we asked participants about WYZ acceptability using the System Usability Scale (SUS), with scores ranging from 0 to 100 and scores >68 being considered above average [31,32]. A threshold of 70% or greater satisfaction on all questions was used to determine acceptability.

#### HIV and Psychosocial Outcomes

HIV and psychosocial outcomes were measured at baseline and 6 months. These included self-reported HIV viral load (detectable or undetectable) [33], self-reported ART adherence [34], depression (Patient Health Questionnaire-9 [PHQ-9]) [35], resilience [36], social provisions [37], social isolation (Patient-Reported Outcomes Measurement Information System [PROMIS]) [38], health care empowerment [39], and unmet subsistence needs and instrumental support [40].

### Data Analysis

Descriptive statistics of the baseline demographics of WYZ study participants were calculated. Next, we examined descriptive statistics for feasibility metrics and compared them to predefined benchmarks. We then calculated frequencies for all of our acceptability metrics measured at the exit survey. Lastly, frequencies for HIV and psychosocial outcomes were calculated at baseline and the exit survey (6 months). For these data evaluated at both baseline and 6 months, we compared data from those who were retained in the study until 6 months and the entire group to examine divergent results. Given that this was a pilot study with limited statistical power, based on guidance from the National Institutes of Health and literature regarding wide confidence intervals and instability of effect sizes from pilot studies, tests of statistical significance and the efficacy of the intervention to compare HIV clinical outcomes preintervention and postintervention were not evaluated [41-44]. All analyses were completed using SAS 9.2 (SAS Institute).

## Results

### Demographics

Demographics of the study participants are presented in Table 1. At baseline, there were 79 participants (mean age 26.9 years, SD 2.9 years). Among the 79 participants, 69 (87%) identified as male, 48 (61%) identified as gay, 33 (43%) identified as Latino, and 16 (21%) identified as Black. Although nearly 57% (45/79) of participants were working, financial insecurity was relatively common, as 62% (49/79) of participants noted “barely getting by” or “not getting by” on the money they have.

Feasibility metrics are presented in Table 2. Of the 92 individuals who were screened, 84 were eligible. Of these 84 individuals, 79 (94%) consented to participate in the study, and 69 (87%) of those who enrolled completed the exit survey at 6 months. All predefined benchmarks were met (Table 2), except for mean minutes in the app per week (benchmark=15 min/week, actual=8.7 min/week). The mean number of logins per week was 5.3 (SD 5.6). In My Health, ART adherence tracking was conducted in 57.9% (5442/9393) of the inquiries. Moreover, the mean number of postings of chat topics on the My Community chat per person per week was 4.8 (range, 1-42), and the number of reported app crashes was less than once per week (0.24).

**Table 1:** Baseline demographics of the study participants.

Demographic		Value^a^ (N=79)
Age (years), mean (SD)		26.9 (2.9)
**Sex at birth, n (%)**		
	Male	69 (87)
	Female	9 (11)
	Decline to answer	1 (1)
**Sexual identity, n (%)**		
	Gay	48 (61)
	Heterosexual	8 (10)
	Other	23 (16)
**Race/ethnicity, n (%)**		
	Asian	3 (4)
	Black	16 (21)
	Latino	33 (43)
	Other	13 (17)
	White	11 (15)
**Financial security, n (%)**		
	I have enough money to live comfortably	24 (30)
	I can barely get by on the money I have	40 (51)
	I cannot get by on the money I have	9 (11)
	Decline to answer	6 (8)
**Work status, n (%)**		
	Full time	28 (35)
	Part time	17 (22)
	Not working	28 (36)
	Other	4 (5)
	Decline to answer	2 (3)

^a^Missing n ranged from 0 to 6 for each item.

**Table 2:** Feasibility metrics, prespecified threshold for each metric, and actual outcome.

Metric and threshold			Actual value
**General**			
	Screened, N	92	
	Eligible (N=92), n (%)	84 (91)	
	Recruited (N=84) (threshold: ≥70%, target N=80 [ie, ≥56]), n (%)	79 (94)	
	Logins (threshold: 1 login/week), mean (SD)	5.3 (5.6)	
	Minutes in the app (threshold: 15 min/week), mean (SD)	8.7 (5.0)	
**My Health**			
	ART^a^ adherence tracking (threshold: ≥3 times/week [ie, ≥43%]), mean/week	58%	
**My Community**			
	Post chat topic (threshold: 1 chat topic/person/week), mean number/person/week (range)	4.8 (1-42)	
**Administrative**			
	Reported app crashes (threshold: <1 app crash/week), mean number/week	0.24	

^a^ART: antiretroviral therapy.

Acceptability metrics are presented in Table 3. Among the 69 participants who completed the study, 77% (n=53) rated their overall experience with the app as excellent to very good, 91% (n= 63) reported the app to be extremely to somewhat easy to download and install, and 96% (n=66) reported that WYZ setup was extremely to somewhat easy. All participants reported being extremely to somewhat comfortable with the security, privacy, and anonymity of WYZ. Moreover, approximately 83% (n=57) stated that they would be extremely to somewhat likely to continue to use WYZ and 94% (n=64) were extremely to somewhat likely to participate in a similar study in the future. Furthermore, 86% (n=59) of participants rated their overall experience with participation in the WYZ study as excellent to very good and 90% (n=62) reported excellent to very good experience with participating in a completely remotely conducted study. The mean SUS score was 75.6, which is considered to be well above average.

HIV and psychosocial metrics are presented in Table 4. At baseline and 6 months, 9% (7/79) and 4% (3/69) of participants, respectively, reported a detectable HIV viral load. During this time, self-reported ART adherence was unchanged. From baseline to 6 months, participants reporting mild depressive symptoms decreased by 9% (30/79, 38% to 19/66, 29%). Moreover, the mean social isolation score decreased by 12.1 points. Overall, we did not note divergent patterns with regard to the HIV and psychosocial metrics between baseline data from the entire sample (N=79) and those who were retained until 6 months (N=69).

**Table 3:** Acceptability metrics exit survey findings.

Metrics		Value^a^ (N=69), n (%)
**How would you rate your overall experience with the WYZ app?**		
	Excellent to very good	53 (77)
	Good	0 (0)
	Fair	2 (3)
	Poor to very poor	14 (20)
**How easy or difficult was it to download and install WYZ on your phone?**		
	Extremely to somewhat easy	63 (91)
	Extremely to somewhat difficult	6 (9)
**How easy or difficult was it to setup WYZ (ie, create a personal passcode, set med reminders, enter your pharmacy information, etc)?**		
	Extremely to somewhat easy	66 (96)
	Extremely to somewhat difficult	3 (4)
**How satisfied were you with the visual design of WYZ?**		
	Extremely to somewhat satisfied	56 (81)
	Extremely to somewhat unsatisfied	12 (19)
**Overall, how helpful was the My Health module in supporting your ability to take your medications as prescribed?**		
	Extremely to very helpful	54 (81)
	Moderately helpful	7 (10)
	A little to not at all helpful	6 (9)
**How satisfied were you with medication and refill reminders?**		
	Extremely to somewhat satisfied	50 (78)
	Extremely to somewhat unsatisfied	14 (22)
**How satisfied were you with the adherence calendar?**		
	Extremely to somewhat satisfied	57 (88)
	Extremely to somewhat unsatisfied	8 (12)
**How satisfied were you with the lab visualization tools (ie, CD4 graph and viral load graphs)?**		
	Extremely to somewhat satisfied	41 (82)
	Extremely to somewhat unsatisfied	9 (18)
**Overall, how helpful was the My Team module in supporting your ability to seek and connect to support and services that you need?**		
	Extremely to very helpful	40 (62)
	Moderately helpful	18 (28)
	A little to not at all helpful	7 (11)
**How satisfied were you with the listing of community resources (ie, list of local resources and organizations in your area)?**		
	Extremely to somewhat satisfied	64 (100)
	Extremely to somewhat unsatisfied	0 (0)
**How satisfied were you with the secure messaging feature with your health care team (ie, being able to send a message to your health care team through WYZ)?**		
	Extremely to somewhat satisfied	45 (82)
	Extremely to somewhat unsatisfied	10 (18)
**How would you rate your comfort level with asking your health care providers to support your participation in the study by agreeing to be added to My Team?**		
	Extremely to somewhat comfortable	51 (88)
	Extremely to somewhat uncomfortable	7 (12)
**Overall, how helpful was the My Community module in supporting your ability to feel connected to other youth living with HIV?**		
	Extremely to very helpful	34 (52)
	Moderately helpful	20 (30)
	A little to not at all helpful	12 (15)
**How satisfied were you with the news feature within WYZ (ie, being able to see the latest HIV and health news in WYZ)?**		
	Extremely to somewhat satisfied	52 (84)
	Extremely to somewhat unsatisfied	10 (16)
**How satisfied were you with the private calendar (ie, being able to add appointments and community events within WYZ)?**		
	Extremely to somewhat satisfied	44 (70)
	Extremely to somewhat unsatisfied	19 (30)
**Overall, how helpful was the private calendar in supporting your ability to keep your health appointments?**		
	Extremely to very helpful	35 (56)
	Moderately helpful	14 (20)
	A little to not at all helpful	13 (23)
**What is your comfort level with the security, privacy, and anonymity provided by WYZ?**		
	Extremely to somewhat comfortable	67 (100)
	Extremely to somewhat uncomfortable	0 (0)
**How likely are you to continue to use WYZ after your participation in the study ends?**		
	Extremely to somewhat likely	57 (83)
	Extremely to somewhat unlikely	12(17)
**In the future, how likely would you be to participate in a similar study where you are asked to use a mobile health app like WYZ on a regular basis?**		
	Extremely to somewhat likely	64 (94)
	Extremely to somewhat unlikely	4 (6)
**How would you rate your overall experience with participation in the WYZ study?**		
	Excellent to very good	59 (86)
	Good	10 (15)
	Fair	0 (0)
	Poor to very poor	0 (0)
**How would you rate your experience participating in a study where everything was conducted remotely (ie, you did not have to come into a clinic or office to complete study activities)?**		
	Excellent to very good	62 (91)
	Good	5 (7)
	Fair	1 (1)
	Poor to very poor	0 (0)
**How would you rate your experience with having to schedule and complete regular check-ins over the phone with a study coordinator?**		
	Excellent to very good	56 (82)
	Good	10 (15)
	Fair	2 (3)
	Poor to very poor	0 (0)
**How would you rate your experience with receiving compensation for your study participation using a reloadable debit card (ie, ClinCard)?**		
	Excellent to very good	60 (87)
	Good	6 (9)
	Fair	3 (4)
	Poor to very poor	0 (0)
**How easy or difficult was it to remember to use WYZ regularly for the 6 months of your study participation?**		
	Extremely to somewhat easy	53 (77)
	Extremely to somewhat difficult	16 (23)
**How helpful was the communication with study staff?**		
	Extremely to very helpful	66 (96)
	Moderately helpful	2 (3)
	A little to not at all helpful	1 (1)

^a^Missing n ranged from 0 to 14 for each item.

**Table 4:** HIV and psychosocial outcomes at baseline and 6 months.

Outcome		Baseline (N=79)	Baseline (N=69)^a^	6 months (N=69)
**HIV viral load, n (%)**				
	Detectable	7 (9)	6 (9)	3 (4)
	Undetectable	70 (89)	59 (88)	60 (87)
	Do not know	2 (3)	2 (3)	2 (3)
	Decline to answer	0 (0)	0 (0)	4 (6)
ART^b^ adherence, mean (SD)		85.0 (16.6)	86.5 (15.3)	85.0 (16.9)
**Depression (PHQ-9^c^), n (%)**				
	No to minimal depression (0-4)	22 (28)	17 (25)	26 (39)
	Mild depression (5-9)	30 (38)	26 (38)	19 (29)
	Moderate depression (10-14)	12 (15)	10 (15)	12 (18)
	Severe to moderately severe depression (15-27)	15 (19)	14 (21)	9 (14)
Resilience, mean (SD)		3.6 (0.7)	3.5 (0.8)	3.6 (0.7)
Social support, mean (SD)		37.9 (6.0)	23.1 (5.3)	37.7 (7.6)
Social isolation, mean (SD)		50.0 (10.0)	35.4 (12.3)	37.9 (7.0)
Health care empowerment, mean (SD)		17.4 (3.1)	18.6 (3.3)	17.4 (3.3)
Relationship with health care provider, mean (SD)		1.4 (0.6)	1.4 (0.6)	1.40 (0.6)
Unmet subsistence needs and instrumental support score, mean (SD)		0.61 (1.2)	0.65 (1.2)	0.67 (1.2)
**Unmet subsistence needs and instrumental support, n (%)**				
	0	54 (70)	46 (70)	37 (65)
	1	11 (14)	9 (14)	11 (19)
	2	6 (8)	4 (6)	3 (5)
	≥3	7 (8)	7 (11)	6 (11)

^a^Baseline data for participants who were retained until the end of the study (6 months).

^b^ART: antiretroviral therapy.

^c^PHQ-9: Patient Health Questionnaire-9.

## Discussion

### Principal Findings

The use of WYZ was highly feasible and acceptable among youth and young adults living with HIV in the San Francisco Bay Area. We met predefined benchmarks for recruitment, mean logins per week, tracking ART adherence, posting chat topics, and app crashes reported. The ease of app download, installation, and setup, and the overall comfort with security, privacy, and anonymity were highly rated. Additionally, participants reported high satisfaction for a research project that was remotely conducted. These findings demonstrate high potential for uptake and app functionality, indicating a promising role for WYZ as an intervention for engagement in HIV care and ART adherence among youth and young adults living with HIV.

Participants used the app for shorter timeframes than were predefined; however, our predefined benchmark may have been an overestimate. Additionally, due to the ability to log ART adherence using out-of-app notifications, some of the interactions with WYZ were not captured in the analytical tools used. In the next phase of this study, we will ask participants to further elaborate about app use during exit qualitative interviews.

Small changes in self-reported HIV and psychosocial metrics from baseline to 6 months highlight the limitations of pilot studies, in which examination of the intervention’s “preliminary impact” is not meaningful due to wide confidence intervals [41-44]. However, we noted improvements in the social isolation score, which, along with the high level of activity in the My Community Chat section, underscore the importance of this feature and deserve further evaluation in future research.

There are currently few mHealth apps in the early stages of development and pilot testing for enhanced engagement in HIV care, ART adherence, and communication with health care teams for people living with HIV [14-17]. The limitations of some of these mHealth apps include lack of specification of a theoretical framework, limited feasibility and acceptability metrics with no predefined benchmarks, small sample size (N<30), wide age range (≥18 years), and availability for either iOS or Android (not both). We have previously summarized these studies [18]. In developing and pilot testing WYZ, we have addressed these limitations.

In this pilot study, we were able to recruit a diverse group of participants with regard to race/ethnicity; however, participants were mainly gay cis-gender men. The other limitations of our study include a single-arm design (ie, no control group) and a relatively small convenience sample of participants from the San Francisco Bay Area who had access to a smartphone and most of who had an undetectable HIV viral load; therefore, study findings may not be generalizable to other populations. The loss to follow-up was approximately 13%, which is lower than estimates among youth and young adults living with HIV in the HIV Research Network (20%) [45] and in other studies in this population (up to 55%) [46]. We believe that the relatively low loss to follow-up may have been due to the fact that this research was conducted completely remotely, which allowed for flexibility for participation. Since the completion of this pilot study, we have resolved all minor bugs and smartphone compatibility challenges. Additionally, we are updating My Health for those who may use long-acting injectables in the near future and the My Team resources section based on user geolocation.

### Conclusion

Youth and young adults living with HIV represent a population that is disproportionately impacted by HIV and requires tailored youth-friendly interventions. There is a dearth of technology-based interventions that address the changing needs of youth and young adults living with HIV. In future research, we will examine the efficacy and effectiveness of WYZ in improving engagement in HIV care and ART adherence among a larger sample of youth and young adults living with HIV taking into account findings from this study. Given the speed of technological advancement and the need for evidence-based solutions for improved HIV health outcomes among youth and young adults living with HIV, we believe that more funding should be allocated to technology-based interventions to move the National Institutes of Health’s Behavioral and Social Sciences Research agenda forward.

## Abbreviations

ART: antiretroviral therapy
HCD: human-centered design
mHealth: mobile health
SUS: System Usability Scale
UCSF: University of California, San Francisco
YAP: Youth Advisory Panel
